# Basic electronic health record (EHR) adoption in ^**^Türkiye is nearly complete but challenges persist

**DOI:** 10.1186/s12913-023-09859-w

**Published:** 2023-09-14

**Authors:** İlker Köse, Sinem Cece, Songül Yener, Senanur Seyhan, Beytiye Özge Elmas, John Rayner, Şuayip Birinci, Mustafa Mahir Ülgü, Esra Zehir, Berrin Gündoğdu

**Affiliations:** 1 Department of Computer Engineering, Alanya University, Saraybeleni St., No:7, Antalya, Turkey; 2 Sağlık 4.0 Company, İstanbul, Turkey; 3 Department of Healthcare Management, İstanbul Medipol University, İstanbul, Turkey; 4 HIMSS Analytics for Europe and Latin America, Leipzig, Germany; 5 Ministry of Health, Ankara, Turkey

**Keywords:** Electronic medical record, EHR adoption, Health informatics, HIMSS, EMRAM

## Abstract

**Background:**

The digitalization studies in public hospitals in Türkiye started with the Health Transformation Program in 2003. As digitalization was accomplished, the policymakers needed to measure hospitals’ electronic health record (EHR) usage and adoptions. The ministry of health has been measuring the dissemination of meaningful usage and adoption of EHR since 2013 using Electronic Medical Record Adoption Model (EMRAM). The first published study about this analysis covered the surveys applied between 2013 and 2017. The results showed that 63.1% of all hospitals in Türkiye had at least basic EHR functions, and 36% had comprehensive EHR functions. Measuring the countrywide EHR adoption level is becoming popular in the world. This study aims to measure adoption levels of EHR in public hospitals in Türkiye, indicate the change to the previous study, and make a benchmark with other countries measuring national EHR adoption levels. The research question of this study is to reveal whether there has been a change in the adoption level of EHR in the three years since 2018 in Türkiye. Also, make a benchmark with other countries such as the US, Japan, and China in country-wide EHR adoption in 2021.

**Methods:**

In 2021, 717 public hospitals actively operating in Türkiye completed the EMRAM survey. The survey results, deals with five topics (General Stage Status, Information Technology Security, Electronic Health Record/Clinical Data Repository, Clinical Documentation, Closed-Loop Management), was reviewed by the authors. Survey data were compared according to hospital type (Specialty Hospitals, General Hospitals, Teaching and Research Hospitals) in terms of general stage status. The data obtained from the survey results were analyzed with QlikView Personal Edition. The availability and prevalence of medical information systems and EHR functions and their use were measured.

**Results:**

We found that 33.7% of public hospitals in Türkiye have only basic EHR functions, and 66.3% have extensive EHR functions, which yields that all hospitals (100%) have at least basic EHR functions. That means remarkable progress from the previous study covering 2013 and 2017. This level also indicates that Türkiye has slightly better adoption from the US (96%) and much better than China (85.3%) and Korea (58.1%).

**Conclusions:**

Although there has been outstanding (50%) progress since 2017 in Turkish public hospitals, it seems there is still a long way to disseminate comprehensive EHR functions, such as closed-loop medication administration, clinical decision support systems, patient engagement, etc. Measuring the stage of EHR adoption at regular intervals and on analytical scales is an effective management tool for policymakers. The bottom-up adoption approach established for adopting and managing EHR functions in the US has also yielded successful results in Türkiye.

## Background

An Electronic Health Record (EHR) is defined as “an electronic recording of patients’ health information created during the provision of health services in healthcare organizations [1]. As per International Organization for Standardization’s (ISO) definition, an EHR means a data repository that allows to store patient data in digital form securely and can be accessed by more than one authorized user. The EHR contains information about the past, present, and future [2, 3]. The primary purpose of the EHR is to support the continuous, efficient, and quality delivery of health services and care [4]. The EHR describes practices for routing and processing any information in electronic systems to provide healthcare-related services to an individual [5].

The first step in the transition to the EHR is digitalization. One of the benefits acquired when switching from a paper-based system to an electronic medium is the rapid access to patient’s health data in electronic media. In addition, the EHR has a positive potential to improve the quality of care and reduce costs in healthcare service delivery. When health data is shared electronically, it facilitates the safe communication of patient information on national and international platforms [6–8].

### Conceptual background

The literature includes many studies on the positive effects of an EHR/ Electronic Medical Record (EMR) on healthcare service quality in adopting its functions, such as closed-loop drug administration, e-order, clinical documentation, etc. For example, a study conducted by Zhou et al. in 2009 established that the use of EHRs improves clinical decisions, facilitates communication with patients, provides faster and more accurate access to medical records, and reduces medication errors [9]. It is seen that the widespread use of EHR increases the quality of health services, reduces errors in medical records, positively affects the working conditions of health workers [10, 11], reduces costs, and reduces medical errors due to the decrease in paper use [12].

The study by Hak et al. in 2020 investigated the benefits acquired if the Open EHR model was adopted in hospitals in Portugal. It was concluded that adopting an EHR is more beneficial when it is used with its functions that are not sufficient on their own [13]. Similarly, a comprehensive review study published by Kose et al. in 2022 examined the publications referring to the benefits of an EHR and found that the EHR alone does not provide a significant benefit for healthcare quality. However, the meaningful use or adoption of an EHR contributes to healthcare quality and patient safety [14].

Our study aims to reveal whether there has been a change in the level of adoption of EHR in Turkey in the three years since 2018 by making comparisons with other countries. It has two main contributions to the literature, as it explores the adoption of the EHR across the country. First, studies measuring country-wide adoption of the EHR are very few. This is due to different measurement methods, costs, ethical clearances, etc., reasons [15]. This study uses the method developed by Jha to establish a comparison with the US, China, and other countries. Thus, it allows the comparison of study results between countries. The second contribution is that it shows the change or progress in the level of adoption of the EHR in the three years since the only study was conducted in Turkey in 2018.

### Country-wide EHR adoption studies

As the adoption and use of an EHR observably has positive contributions to the quality of healthcare services and patient safety, measuring the stage of EHR adoption throughout the country has become an important management tool for the health policymakers of the countries. As such, researchers and ministries of health conducted numerous studies measuring the stage of EHR adoption across the country. Studies conducted in the last 15 years show that the EHR adoption stage is increasing in the US, Türkiye, Japan, Norway, the United Kingdom, Saudi Arabia, China, Brazil, France, and Russia [16].

The number of studies measuring the nationwide adoption of the EHR is very few in the literature. In this situation, measurability difficulty, ethical permissions, costs, data access difficulty, etc., are considered to be due to reasons such as. Our study provides the opportunity to compare the study data with other countries. It contributes to the literature, as it addresses the level of adoption of the EHR throughout the country. This study is one of the rare studies in the world that measures EHR adoption across the country. Its main contribution to the literature is to show the change or progress in the level of EHR adoption in three years since the only study was conducted in Türkiye in 2018. Another contribution of our study is that the measurement models used are different, and the differences in survey questions and perspectives make it difficult to compare countries. This study uses the method developed by Jha to create a benchmark with the US, China, and other countries. Thus, it allows to compare the results of the study between countries.

The first study we could access in this respect was carried out by Jha et al. in 2009. Within the scope of the study, 24 functions of EHR adoption of hospitals operating in the US were measured. The results obtained from the study were divided into two: basic EHR functions and comprehensive EHR functions. While basic EHR functions were limited to one clinic for clinical documentation, Computer Physician Order Entry (CPOE), Clinical Decision Support System (CDSS), and laboratory and imaging results, comprehensive EHR functions were observed to be available for use in all clinics of the hospital. This study demonstrated that 1.5% of the hospitals operating in the US have comprehensive EHR functions, and 7.6% have basic EHR functions [17, 18]. The study conducted by Jha et al. in 2010 revealed the barriers to reducing paper use and adopting the use of EHRs in the US. Based on the study conducted by Jha et al. in 2011, it was observed that hospitals with an electronic health record system increased from 15.1% to 2010 to 26.6% in 2011, and hospitals with a comprehensive EHR system increased from 3.6 to 8.7% [19, 20].

Jha et al. conducted a study in 2014 and comparatively discussed the stage of EHR adoption in the US and the UK. In addition, they compared the methodology applied by both the US and the UK for EHR adoption. Accordingly, it was understood that the US carried out a bottom-up adoption while the United Kingdom carried a top-down adoption. When the results are assessed, it is considered that the management through the bottom-up adoption implemented by the US is more effective [21]. In their study during the same year, however, Johnson et al. responded to this criticism by emphasizing that the rate of EHR adoption in the UK was almost 100% in the primary healthcare field at the time when the rate of EHR adoption in the US was 10–30%. A study by Wilson and Khansa in 2018 shows that both the US and the UK face significant hurdles in establishing their countrywide EHR systems and implementing their visions while EHR implementation and adoption are on the rise in both countries [22].

Similar studies were conducted by Adler-Milstein et al. on hospitals in the US in 2014, 2015, and 2017 [23–26]. These studies prove that hospitals with extensive EHR functions grew in number over time in the US. The EHR adoption rates in 2014, 2015, and 2017 were 25.5%, 34.1%, and 39.1%, respectively. In addition, the rate of hospitals with basic EHR functions rose over time, which was 58.9% in 2014, 44.1% in 2015, and 41.4% in 2017.

The study by Hu et al. in 2020 investigated the relationship between the characteristics of psychiatric hospitals in the US and the adoption of EHR. In the Method section, a criterion has been determined whether or not to have a certificate. If the hospitals carry out technology-based operations in the care service provided to the patient, they are accepted as certificate holders. It is considered not a certificate holder if it carries out these processes mostly on paper. The location, size, and type of hospital (government, for-profit, not-for-profit) influence EHR adoption. It has been observed that 47.4% of psychiatric hospitals holding EHR certificates in the USA have adopted the EHR. A link has been established between the 95% adoption of large hospitals and their certification [27].

Korea is the other country where intensive efforts are used to adopt EHRs. The initial study published by Yu et al. in 2003 examined the computer usage rates among physicians while doing clinical documentation, and it was understood that 98% of physicians were very open-minded about adopting digitalization processes by performing electronic documentation [28]. The study by Woong Park et al. in 2005 investigated the prevalence of EHR use and the use of CPOE in general and teaching hospitals. It was observed that the use of CPOE was 80.3%, while it was concluded that the use of a complete EHR is 9% [29]. In a study conducted by Yoon et al. in 2012, EHR adoption in general and teaching hospitals was found to be 37.2% [30]. On the other hand, a study by Kim et al. in 2017 established that the percentage of hospitals with basic EHR functions in teaching and general hospitals in Korea was 58.1% [31].

The other study on the adoption of EHR was carried out by Otieno et al. in 2008. This study measured the effectiveness of EHR systems in Japan. It was observed that the stage of EHR systems adoption in hospitals is 30% [32]. The study by Pereira et al. in 2020 elaborated on the progress of digital transformation in Portugal. It was concluded that digital transformation accelerated and was adopted faster over time [33]. In a study by Shu et al. in 2014, EHR adoption of EHRs in China’s tertiary care hospitals was evaluated. This study applied a national survey titled EHR Rating Model (MEG), which assigns hospitals a rating from 0 to 7. This study demonstrated that 30.7% of 848 hospitals were Level 0, 12.0% Level 1, 31.7% Level 2, 22.2% Level 3, 2.7% Level 4, 0.6% Level 5 and 0.1% Level 6 [9].

There are studies on the adoption of the EHR broken down by countries and other studies in which countries have compared themselves with other countries. A study comparing the level of EHR adoption between the US and Japan was conducted by Kanakubo and Kharrazi in 2014, which examined the Electronic Health Record adoption trends in both countries. A countrywide hospital survey of Japan was used to obtain EHR adoption rates among Japanese hospitals. Comparable datasets from the Health Information and Management System Society (HIMSS) Electronic Medical Record Adoption Model (EMRAM) and the American Hospital Association (AHA) were used to extract EHR adoption rates among hospitals in the US. The result of the study showed that the US surpassed Japan in 2014 in adopting the EHR for small, medium, and large hospitals [34].

The other study, including a comparison between countries, was carried out by Liang et al. in 2021 to compare the stage of EHR adoption in China and the US. The trends in EHR adoption rates in China and the United States were compared using hospital survey data from the Health Information Management Association (CHIMA), China, for the period between 2007 and 2018, and AHA survey data from the United States for the period between 2008 and 2017. From 2017 to 2018, the level of EHR adoption in China increased from 18.6 to 85.3%, while in the US, from 2008 to 2017, the level of EHR adoption increased from 9.4 to 96%. The EHR adoption rates in China and the US increased significantly over the last ten years [35].

The study conducted by Sadoughi et al. in 2019 showed that a majority of the studies requiring the adoption of EHR mostly preferred the survey method for data collection. In addition, this study argues that adopting EHR systems is influenced by multidimensional, complex, and different types of factors in healthcare organizations [36].

Türkiye has some studies to measure the adoption stage of EHR systems. The initial study in this field was published by Kose et al. in 2020. This study measured the adoption stages of hospitals between 2014 and 2017 and investigated the relationship between adoption stages and hospital size. The study results showed that 63.1% of all hospitals in Türkiye have at least basic EHR functions, and 36% have comprehensive EHR functions. Additionally, it was observed that small hospitals were in a better position to adopt certain EHR functions than large hospitals [14].

On the other hand, our study was conducted to measure the change in the EHR adoption stages of public hospitals in Türkiye after the study published by Kose et al. in 2020. The adoption stage of the EHR on a national scale was measured in line with the survey results.

As Adler-Milstein et al. [23–26], Kanakubo and Kharrazi [34] and Liang et al. [35] did, measuring the adoption level of EHR in a country with intervals using analytical scales can be taken as an effective management tool for policymakers. Similar to those studies, this study aims to see the progress made in the countrywide EHR adoption in Türkiye between 2018 and 2021. Also, the results obtained were compared with the studies conducted in other countries.

## Methods

### Study design

As seen in the literature review, the survey method was used as a measurement tool in studies measuring the level of EHR adoption countrywide. For this purpose, the most frequently used questionnaires were AHA in the US [16–21] and Korea [30], CHIMA in China [34], and HIMSS EMRAM in Türkiye [14]. Similar to our study published in 2020, the HIMSS EMRAM questionnaire was used in this study. To make a benchmark with other countries, as in many studies [16–20, 30, 34], we matched our model (EMRAM levels) with Basic EHR, No EHR, and Comprehensive EHR levels made by Jha [16].

### Study setting

**Fig. 1:**
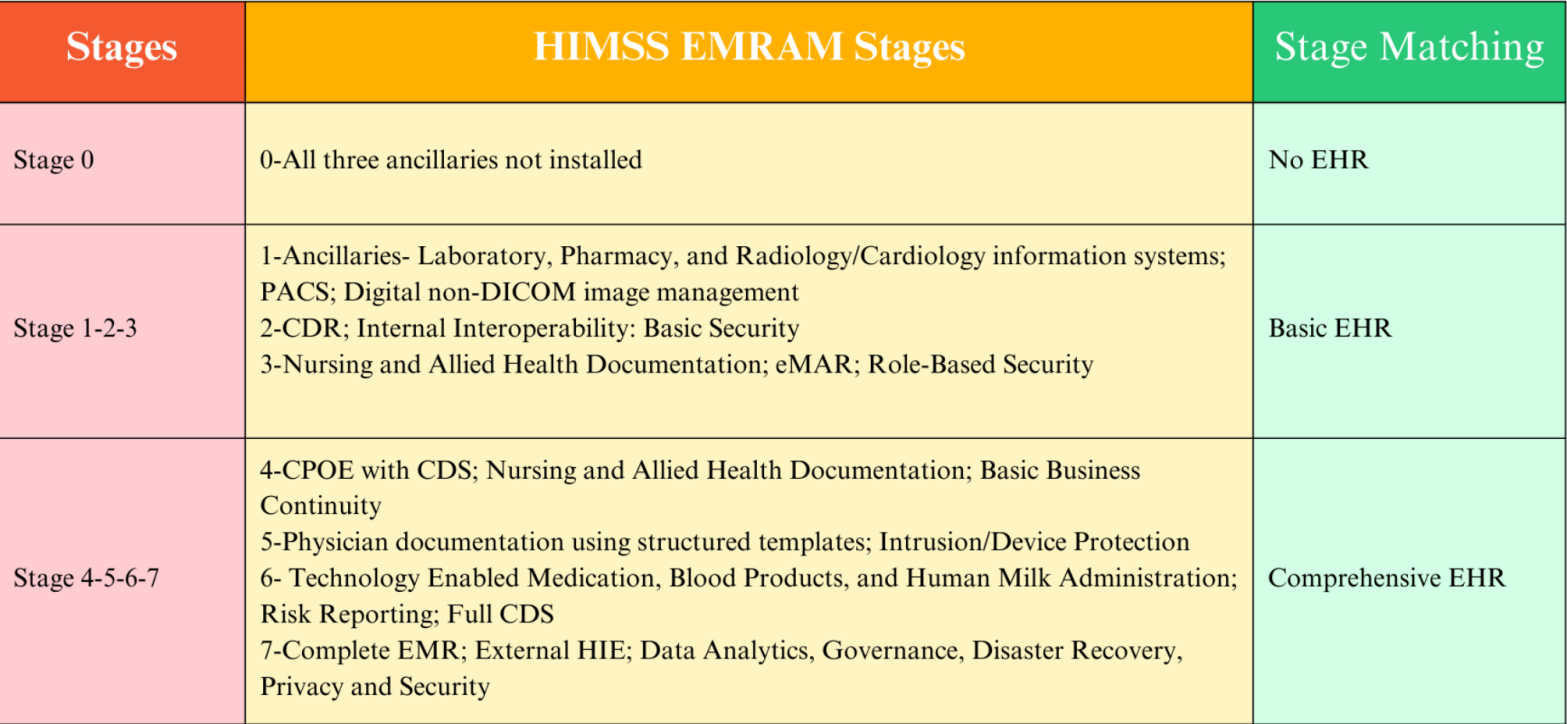
Matching Stages

It is understood that the studies measuring the EHR adoption on a national scale use models, such as CHIMA, AHA, and HIMSS EMRAM. To make a more accurate comparison, the data for this study was collected by the authors with the HIMSS EMRAM survey, which we used in our study published in 2020. HIMSS EMRAM is an eight-stage (0–7) model that measures the adoption and use of EHR functions (Fig. 1). The datasets generated and analyzed during the current study are not publicly available due to MoH regulations but are available from the corresponding author upon reasonable request. This study was approved by the Medical Ethics Committee of Istanbul Medipol University of Medical Sciences A survey is used as a measurement tool. Based on the survey results, healthcare organizations are assigned a score that goes up to 7 according to the stage of EHR adoption. In the study conducted by Furukawa and Pollack in the US in 2020, it was concluded that the hospitals, which were validated as HIMSS EMRAM and O-EMRAM Stage 6–7, being certified for HIMSS EMRAM were influential in the adoption of EHR systems and significantly helped the nurse group to adopt such systems [37].

### Aim of the study

Our study aimed to have the HIMSS-EMRAM survey filled out by 717 state hospitals in Türkiye between April 2021 and December 2021. After the survey, the EMRAM Gap Analysis report created by HIMSS Analytics was examined and evaluated. This report consists of 5 main topics: 1) “General Stage Status, 2) Information Technologies Security, 3) Electronic Health Record/Clinical Data Store, 4) Clinical Documentation, and 5) Closed Loop Product Management. As in the General Stage Status, the staging was performed between 0–7 for other topics. Thus, a detailed examination was presented regarding the general stages of hospitals and the situation covered by other topics.

### Comparison of study data

To compare the survey results with those from other countries, the EMRAM stages were first associated with the following progressions: No EHR, Basic EHR, and Comprehensive EHR (Jha et al., 2009), developed by Jha et al. in 2008. Additionally, this match-up is in line with the studies conducted by Kanakubo & Kharrazi, Rae Woogn Park et al. in the US, Japan, and Korea and allows the data obtained to be compared [29, 34].

Since this study relies on the EMRAM survey as the data source, a stage assessment was made on the “Adoption of Electronic Health Record”. The concept of “adopting” discussed here means that EHR functions exist and are used within the hospital. This position was confirmed through observations, semi-structured interviews with healthcare professionals, and discussion groups during the survey completion process. In addition, the hospitals scored as Stage 6 and 7 were visited, and it was validated that those hospitals were in practice Stage 6 or 7, with studies lasting 1 to 2 days. The data obtained from the survey results were analyzed with QlikView Personal Edition. Thus, the data were visualized and analyzed in detail.

## Results

The surveys found to be inconsistent or of poor quality by the HIMSS Analytical evaluation system were excluded from this study. If multiple surveys were filled out in the same hospital during the year, only the most recent survey was included in the analysis.

### General stage status

A survey was sent to all 717 state hospitals operating in Türkiye. The number of hospitals that completed the survey (89.4%) was 641. However, (10.6%) 76 hospitals were not included in the study due to the inconsistency of the survey data. The number of hospitals in Türkiye as of 2021 [38] and the ratios of the hospitals that filled out the survey is given in Table 1.

**Table 1:** The Survey Completion Rate by the Type of Hospitals

Hospital Type	Specialty Hospitals	General Hospitals	Teaching and Research Hospitals	Total
**Number of Hospitals**	36	586	95	**717**
**Number of Surveys Filled Out**	35	512	94	**641**
**Survey Fill Rate**	97.2%	87.3%	98.9%	**89%**

In line with the survey results obtained based on the responses to the survey given by the hospitals, the general stage distribution of EMRAM is shown in Table 2. Given the type of hospitals that filled out the survey, the survey results include no stage “0” hospital. According to the stage match-up in Fig. 1, any type of hospital adopt and use at least one of the EHR functions. The rate of hospitals with basic EHR functions and comprehensive EHR procedures for specialty hospitals is 77.1% and 22.9%, respectively. The rate of hospitals with basic and comprehensive EHR functions for general hospitals is 65.1% and 34.9%, respectively. The rate of hospitals with basic and comprehensive EHR functions for teaching and research hospitals is 68.1% and 24.5%, respectively.

**Table 2:** General Stage Distribution of Hospitals Filling Out the Survey by Type of Hospitals

	Stage 0	Stage 1	Stage 2	Stage 3	Stage 4	Stage 5	Stage 6	Stage 7	Total
**Specialty Hospitals**		2(5.7%)		25(71.4%)		1(2.9%)	7(20%)		**35 (5.4%)**
**General Hospitals**		24(4.7%)	49(9.6%)	260(50.8%)	42(8.2%)	59(11.5%)	74(14.5%)	4(0.7%)	**512 (79.9%)**
**Teaching and Research Hospitals**		4(4.3%)	6(6.4%)	54(57.4%)	7(7.4%)	9(9.6%)	13(13.8%)	1(1.1%)	**94 (14.7%)**
**Total**		**30 (14.7%)**	**55(16%)**	**339(179.6%)**	**49(15.6%)**	**69(23.9%)**	**94(48.3%)**	**5(1.8%)**	**641 (100%)**

**Fig. 2:**
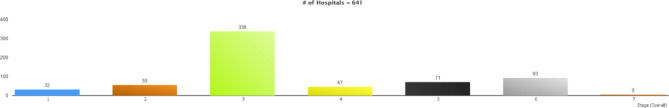
General Stage Distribution of the Hospitals Filling out the Survey

Figure 2 provides the general distribution of EMRAM stages matched according to the survey results of the hospitals. According to this match-up, 425 (66.3%) of the 641 public hospitals that filled out the survey had basic EHR functions, while 216 (33.7%) had comprehensive EHR functions. In this case, it is understood that hospitals with basic EHR functions can fulfill their comprehensive EHR functions by quickly eliminating their deficiencies.

**Fig. 3:**
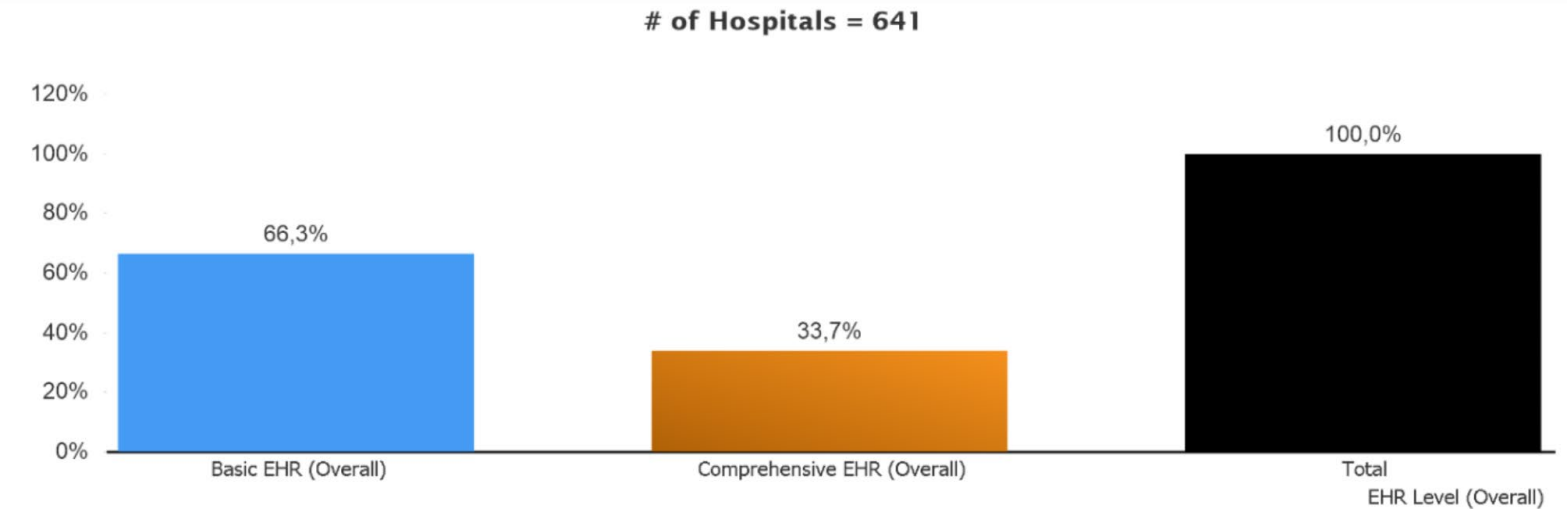
Distribution of the Hospitals Filling Out the Survey by Stage Match-up

The general stage distribution of EMRAM based on the survey results obtained in line with the responses to the survey given by the hospitals is shown in Fig. 3. There is no stage “0” hospital in the survey results for the hospitals that filled out the survey. This result shows that all hospitals adopt and use at least one of the EHR functions. 66.3% of the hospitals are included in the Stage 1-2-3 category. That means these hospitals have basic EHR functions, such as PACS, Emar, and digitalization of nursing documents. 33.7% of the hospitals are included in the Stage 4-5-6-7 category. These hospitals are observed to have extensive EHR functions (Figure 2).

### Information technology security

**Fig. 4:**
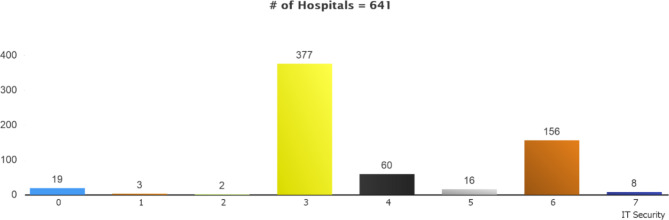
Stage Distribution of the Hospitals Filling Out the Survey by IT Security

Based on the survey results for the hospitals, the distribution of the Information Technologies Security stage is shown in Fig. 4. Hospitals are mostly graded stage 3 concerning Information Technologies Security because they fail to meet the Business Continuity criteria required within the EMRAM criteria. Although hospitals fulfilled other criteria at higher stages, they remained at Stage 3 as this criterion was not met. While, on the other hand, there are 93 hospitals with a general stage of Stage 6, the existence of 156 hospitals in the field of information technology security shows that hospitals do better in terms of security compared to other fields.

### Electronic health record (EHR)/clinical data repository (CDR)

**Fig. 5:**
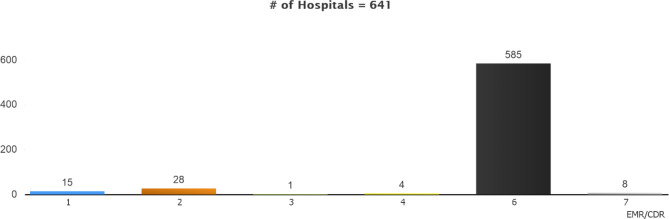
Stage Distribution of the Hospitals Filling Out the Survey by EHR/CDR

Figure 5 shows the EMRAM stages distribution in Electronic Health Record/Clinical Data Repository based on the hospital survey results. It is understood that the hospitals are at a perfect stage regarding Electronic Health Record/Clinical Data Repository. It is possible to state that this results from hospitals filling in health records thoroughly and meticulously.

### Clinical documentation

**Fig. 6:**
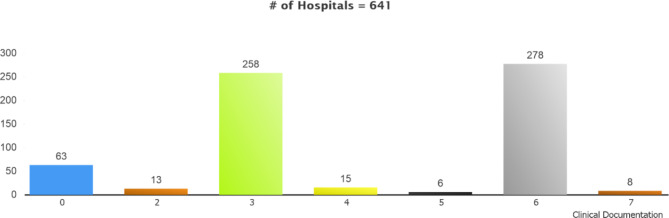
Stage Distribution of the Hospitals Filling Out the Survey by Clinical Documentation

Based on the hospital survey results, the general distribution of EMRAM stages in Clinical Documentation is given in Fig. 6. In this case, the figure shows that all data entry forms in 63 (9.8%) hospitals are not structured yet. Upon review, it was observed that the anamnesis forms in the emergency departments of 63 hospitals did not allow structural data entry yet, while the physician and nurse anamnesis forms in the outpatient and inpatient services were structured in nearly all the hospitals. Therefore, the stage of the hospitals in question was 0 concerning the clinical documentation assessment.

### Closed-loop management (CLMA)

**Fig. 7:**
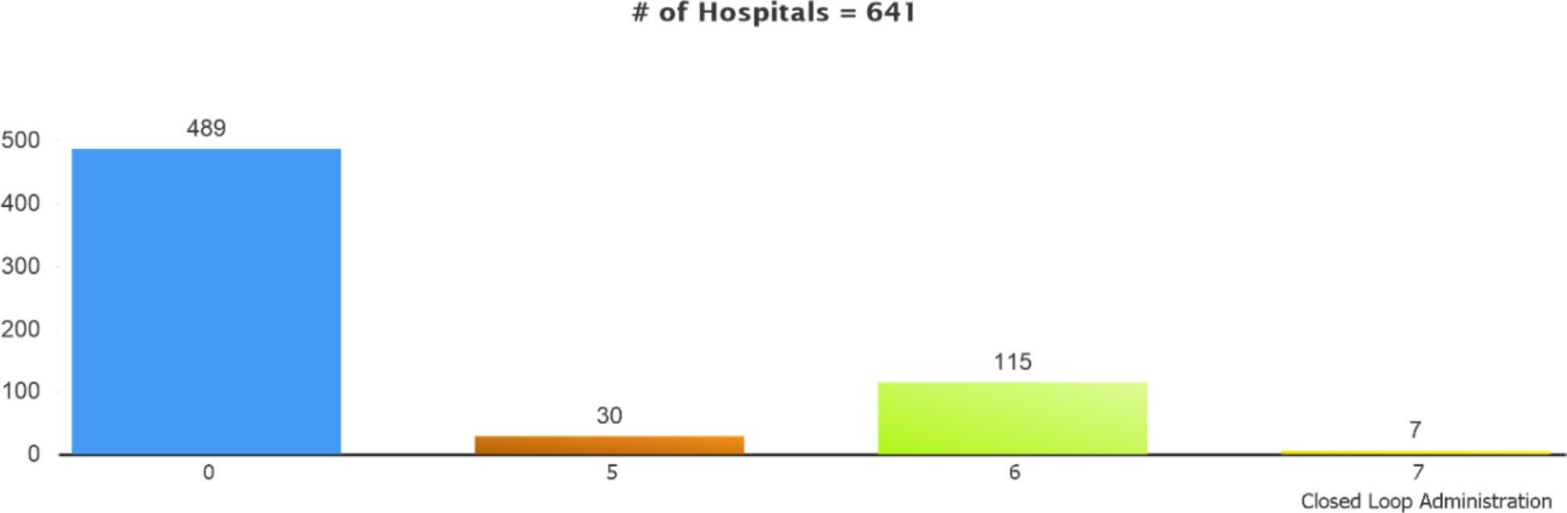
Stage Distribution of the Hospitals Filling Out the Survey by Closed-Loop Management

The distribution of EMRAM stages for Closed-Loop Management based on the survey results for the hospitals is given in Fig. 7. According to the survey results, there are no stage 1-2-3 hospitals under this topic. That is since closed-loop product processes (medicine, blood and blood products, breast milk, sampling, etc.) are mandatory EMRAM criteria of those stages and are not applied in most hospitals. Therefore, it is understood that only 115 (23.5%) hospitals adopted CLMA or were validated for the EMRAM Stage 6 or 7. Closed-Loop Management is never used in hospitals, reaching a rate of 76.5%.

## Discussion

Measuring the adoption stages for EHR functions across the country provides essential information for service providers and policymakers and enables the country’s general situation to be analyzed by comparing it with other countries.

Table 3 includes current and similar studies conducted in recent years. The study conducted by Adler-Milstein et al. in the US between 2010 and 2015 found that 41.4% of all the hospitals operating throughout the country had basic EHR functions, 39.1% had comprehensive EHR functions, and 80.5% had at least one EHR function [39]. A study by Hu et al., which measured the stage of EHR adoption in psychiatric hospitals in the US in 2016, found the rate of hospitals with at least one EHR function to be 47.4% [27]. A similar study conducted by Liang et al. in the US and China between 2007 and 2018 found the rate of hospitals with at least one EHR function to be 96% in the US and 85.3% in China [40]. In another study by Kim et al. in Korea in 2017, the rate of hospitals with basic EHR functions was 46.5%, and the rate of hospitals with comprehensive EHR functions was 11.6%. The rate of hospitals with at least one EHR function is 58.1% [31].

When the study conducted in Türkiye by Kose et al. in 2017 was compared with the studies conducted in the US, China, and Korea, no hospital existed without any EHR function. The rate of hospitals with basic EHR functions was 27.1%, while that of hospitals with comprehensive EHR functions was 36%. The rate of hospitals with at least one EHR function is 63.1% [14]. The study published by Liang et al. in 2021 found the rate of hospitals with at least one EHR function in the US to be 96%. Within the scope of the same study, the rate of hospitals with at least one EHR function in China was 85.3%. In the study by Kose et al., spanning the period from 2014 to 2017, this rate was found to be 63.1% in Türkiye. These studies demonstrate that the US, China, and Türkiye are in leading positions in EHR adoption compared to other countries.

When these studies on the adoption of the EHR are examined, the scales used to show similarity. Adler-Milstein (2017), Kim et al. (2017), and Liang et al. (2021) used the American Hospital Association annual survey as a scale to measure the adoption stage of EHRs across the country. Only Liang et al. (2021) further used the Chinese Health Information Management Association (CHIMA) scale to measure the stage in China. Additionally, the study by Kose et al. (2020) used the HIMSS-EMRAM survey as a scale [30, 34, 40].

**Table 3:** Comparison of Data with Similar Publications

Study Year	Publication Year	Author	Country	Measure	Basic EHR	Comprehensive EHR	At Least Basic EHR (or Overall EHR)
2008–2015	2017	Adler-Milstein et al.	USA	American Hospital Association annual survey	41.4%	39.1%	80.5%
2015	2017	Kim et al. (2017)	Korea	American Hospital Association annual survey	46.5%	11.6%	58.1%
2016	2020	Hu et al.	USA (psychology hospitals)	American Hospital Association annual survey			47.4%
2014–2017	2020	Kose et al. (2020)	Türkiye	HIMSS EMRAM	27.1%	36%	63.1%
2007–2018	2021	Liang et al.	USA	American Hospital Association annual survey			96%
2008–2017	2021	Liang et al.	China	Chinese Health Information Management Association (CHIMA)			85.3%

As part of this study, on the other hand, the rate of hospitals with basic EHR functions was found as 66.3%, and the rate of hospitals with comprehensive EHR functions was 33.7%. When the data from our previous study spanning the period from 2014 to 2017 are compared, it is understood that the number of hospitals with basic EHR functions in Türkiye increased by 50% in the last five years, reaching 100% [14]. The number of hospitals with comprehensive EHR functions decreased by 13.97%. That can be presumed to mainly result from the fact that the HIMSS EMRAM criteria were updated in 2018, becoming more comprehensive. As part of the criteria updated in 2018, it became a necessity for hospitals to apply the criteria to cover 50% of the number of beds, the number of physicians, or the number of wards in order for them to fulfill the HIMSS EMRAM Stage 6 criteria. Back in 2017, it was sufficient for hospitals to apply the HIMSS EMRAM criteria in a single ward. Due to the increase in expectations of EMRAM criteria, the stages of hospitals decreased.

## Conclusion

There are many benefits of adopting EHRs at the hospital or country level. While most studies focus on the implementation and measuring the benefits of EHR, several studies have attempted to examine the factors that facilitate EHR adoption in addition to measuring the level of EHR adoption across the country. These studies have focused on various factors such as hospital size (44,45), policies implemented by governments (46), and adoption methods such as the top-down (47) or bottom-up approach. Those studies indicated that different countries might achieve EHR adoption in different ways. For example, Türkiye’s experience with EHR adoption is like that of the United States [25], which also implemented a bottom-up approach but differed from the UK’s experience [21]. It indicates that each country must plan its EHR adoption studies based on its unique circumstances. Regularly measuring EHR adoption levels using widely accepted scales is critical for policymakers to act promptly and effectively. Therefore, the primary motivation for this and similar studies is to measure EHR adoption levels across all hospitals using widely accepted scales.

In conclusion, our study on EHR adoption in Türkiye suggests that political stability, determination, and targeted actions, such as including EHR adoption as a target in the Strategic Plan of the Ministry of Health in 2013, have been critical drivers of EHR adoption in the country. However, our findings also highlight the need for ministerial support, willingness to invest, and strong clinical leadership to achieve successful outcomes. To ensure effective EHR adoption, policymakers must consider local conditions, and measuring EHR adoption at regular intervals is a valuable management tool. Thus, one of the future studies will definitely be conducting the updated EMRAM 2022 criteria in the same hospitals to compare the results with our previous studies and focus on closed-loop management, information security, and patient-reported outcomes. Additionally, continuous care is another challenging issue in measuring the quality of healthcare given by several facilities in the same region. Thus, as the research group, we aim to develop a novel model measuring several institutions together in EHR adoption. We strongly suggest to researchers study more comprehensive models to measure EHR adoption at both hospital and country levels.

Finally, while it is increasingly common for hospitals to measure quality, efficiency, and EHR adoption using various measurement tools, there needs to be a scale that measures hospitals’ innovation performance. We have ongoing research to develop a novel model to measure hospitals’ innovation and digital transformation performance, which will provide valuable insights for policymakers and healthcare providers.

## Data Availability

The datasets generated and analyzed during the current study are not publicly available due to MoH regulations but are available from the corresponding author upon reasonable request.

## Abbreviations

EHR: Electronic Health Record
EMRAM: Electronic Medical Record Adaption Model
CPOE: Computer Physician Order Entry
CDSS: Clinical Decision Support System
ISO: International Organization for Standardization
EMR: Electronic Medical Record
HIMSS: Health Information and Management System Society
AHA: American Hospital Association
CHIMA: China Health Information Management Association
CDR: Clinical Data Repository
CLMA: Closed-Loop Management
MoH: Ministry of Health
